# Meta‐analysis of laparoscopic transcystic *versus* transcholedochal common bile duct exploration for choledocholithiasis

**DOI:** 10.1002/bjs5.50132

**Published:** 2019-01-23

**Authors:** M. Bekheit, R. Smith, G. Ramsay, F. Soggiu, M. Ghazanfar, I. Ahmed

**Affiliations:** ^1^ Department of Surgery, Aberdeen Royal Infirmary Aberdeen UK; ^2^ Scottish Clinical Research Excellence Development Scheme, Rowett Institute University of Aberdeen Aberdeen UK; ^3^ Department of Surgery Royal Free Hospital London UK

## Abstract

**Background:**

It is not clear whether laparoscopic transcystic exploration (LTCE) laparoscopic choledochotomy (LCD) is superior in the management of choledocholithiasis. In this meta‐analysis, the success of LTCE *versus* LCD was evaluated.

**Methods:**

Cochrane Central Register of Controlled Trials, Web of Science, Trip, PubMed, Ovid and Embase databases were searched systematically for relevant literature up to May 2017. Studies that compared the success rate of LTCE and LCD in patients with choledocholithiasis were included. PRISMA guidelines were followed. Multiple independent reviewers contributed on a cloud‐based platform. Random‐effects model was used to calculate odds ratios (ORs) or standardized mean differences (MDs) with 95 per cent confidence intervals. An *a priori* hypothesis was generated based on clinical experience that LTCE is as successful as LCD.

**Results:**

Of 3533 screened articles, 25 studies comprising 4224 patients were included. LTCE achieved a lower duct clearance rate than LCD (OR 0.38, 95 per cent c.i. 0·24 to 0·59). It was associated with a shorter duration of surgery (MD −0·86, 95 per cent c.i. −0·97 to −0·77), lower bile leak (OR 0·46, 0·23 to 0·93) and shorter hospital stay (MD −0·78, −1·14 to −0·42) than LCD. There was no statistically significant difference in conversion, stricture formation or reintervention rate.

**Conclusion:**

LCD has a higher rate of successful duct clearance, but is associated with a longer duration of surgery and hospital stay, and a higher bile leak rate.

## Introduction

Concomitant common bile duct (CBD) stones are present in 3–15 per cent of patients with symptomatic cholelithiasis in the Western world^1^. In approximately 2 per cent of patients these stones are considered clinically significant^2^. These patients require CBD stone extraction for symptomatic relief and to prevent serious associated complications, including cholangitis, hepatic abscess and acute pancreatitis^3^.

Advances in preoperative imaging, endoscopic and laparoscopic surgical techniques have led to less invasive methods of extracting CBD stones^4^, and there is now a range of potential management options^5^. In the early era of laparoscopic cholecystectomy, patients with suspected CBD stones were commonly referred for endoscopic retrograde cholangiopancreatography (ERCP) and sphincterotomy. Although still a valid management option, this approach has the disadvantage of being a two‐stage procedure with potential increased costs and morbidity^6^. With increased laparoscopic experience, single‐stage laparoscopic cholecystectomy and CBD exploration have become an increasingly popular alternative^7^. There are two main approaches to laparoscopic common bile duct exploration (LCBDE): laparoscopic transcystic exploration (LTCE), reaching the CBD via the cystic duct, and laparoscopic choledochotomy (LCD), exploring the CBD directly via a choledochotomy.

Several high‐quality comparisons between ERCP and LCBDE have been performed^8^. With the trend of primary CBD closure and reduced morbidity of this procedure^9^, as opposed to the resultant morbidity from ERCP^10^, there is a movement towards single‐stage surgical management of gallstone disease. Little attention, however, has been paid to comparing the two different approaches of LCDBE. Frequently, data for both approaches are reported together as combined figures, limiting direct comparison of success rate and safety. Moreover, patients undergoing either of the approaches are treated differently in the postoperative period^11^. The standard surgical approach has been LCD. With increased experience, a trend is observed towards LTCE assuming lower morbidity. Currently, evidence is limited on whether LTCE results in at least a similar clearance rate of the CBD stones. The aim of this study was to compare both approaches in terms of clearance rate and other relevant outcomes from the available literature.

## Methods

This systematic review and meta‐analysis was conducted according to PRISMA guidelines^12^. The study protocol was registered with PROSPERO, the international prospective register of systematic reviews (registration number CRD42017079458).

### Eligibility criteria and outcomes

The criteria for considering studies for inclusion in this review were defined using the Population, Intervention, Comparison and Outcome (PICO) strategy. The study population comprised adults who presented with CBD stones diagnosed via imaging, with no previous cholecystectomy. The type of intervention was LTCE for the treatment of CBD stones, and the comparator was the standard LCD approach. The primary outcome of the study was the success rate of the approach, identified by the rate of complete clearance without conversion from transcystic to transcholedochal or from either to an open approach. The reported incidence of retained CBD stones was used to validate the clearance rate. Secondary outcomes included duration of surgery, length of hospital stay, conversion to open procedure, and intraoperative or postoperative complications (early and late).

Interventional and observational studies comparing the LTCE approach with LCD were evaluated for inclusion. Studies were excluded if they did not meet the above criteria or if there was no statement in the article on ethical approval. Articles that did not report outcomes for both arms, review articles or meta‐analyses, editorials and animal studies were excluded.

The search was primarily for articles in English. Studies presented in other languages were, however, considered for inclusion based on the inclusion and exclusion criteria and the presence of an abstract in English, French or Italian. There was no publication date restriction.

### Literature search

Cochrane Central Register of Controlled Trials, Web of Science, Trip, PubMed, Ovid and Embase databases were searched systematically to identify relevant articles published up to May 2017. Citation alerts were set up for potentially missed or recent articles published during the manuscript synthesis. Google and Google Scholar were used to find non‐indexed publications, to reduce the risk of publications bias.

### Study selection and data extraction

Four authors conducted their database search independently. They screened titles and abstracts first. Duplicates were handled in Mendeley® (Elsevier, London, UK)^12^. If more than one paper was published by the same group, their most recent publication was selected if the number included was larger than their earlier publication and there was no clear indication that the recent study did not include patients from the earlier publication. Articles found suitable for inclusion were then cross‐referenced to ensure inclusion of all eligible studies. Articles that could not be obtained from the internet with multi‐institutional access were sought via the library service. Detailed search strategies, Boolean operators, different search techniques, filters and limits were documented (*Table* S1, supporting information).

### Platforms used for collaborative work

The independently short‐listed search results from across the databases were exported to cloud‐based shared tables (Google sheets: access https://docs.google.com/spreadsheets/d/1uswlPUDrrX9BQj_VpSEHpiQKk91y6Gq1av4SGZ4JYEg/edit?usp=sharing) for further selection and conflict resolution. Two authors decided on conflicts in inclusion or exclusion. Included studies were further exported to Mendeley® citation manager for citations. The initial manuscript draft was produced on Google Docs (Google, Mountain View, California, USA) for live collaborative editing, then exported to Word® processor (Microsoft, Redmond, Washington, USA) for final editing.

### Quality assessment

The median quality score for the RCTs was judged based on the Cochrane Handbook^13^. The quality assessment stratifies the current evidence and projects the need for further research on the topic based on the quality of the available evidence into: high‐quality evidence where further research is not expected to change the current confidence in the estimate of the effect size, moderate‐quality evidence if further research is likely to influence confidence in the estimated effect and may change it; low‐quality evidence if further research is very likely to influence confidence in the estimate of effect and is likely to change it; and very low‐quality evidence when there is no certainty about the estimated effect. The median quality score for the non‐randomized studies was based on the West suggestion^14^. Quality grading is dependent on the rigor of the research methodology.

### Statistical analysis

Statistical analysis was performed using Comprehensive Meta‐Analysis software for Windows® version 2 (Biostat, Englewood, New Jersey, USA). This software was used to generate all forest plots. Heterogeneity was calculated with the χ^2^ test. An *I* ^2^ value above 30 per cent or *P* < 0·050 was considered an indicator of observed heterogeneity^15^. In case of significant heterogeneity, the random‐effects model was used rather than the fixed‐effect model. Results for dichotomous data were stated as odds ratios (ORs), and those for continuous data as standardized mean differences (MDs). Both were provided with their 95 per cent confidence intervals. The random‐effect models was applied for the estimated pooled effect size, given the observed heterogeneity and an adequate number of the included studies^16^.

When data were summarized as median (range) rather than mean(s.d.), these values were converted to mean(s.d.) when necessary, as described previously^17^.

## Results

**Figure 1:**
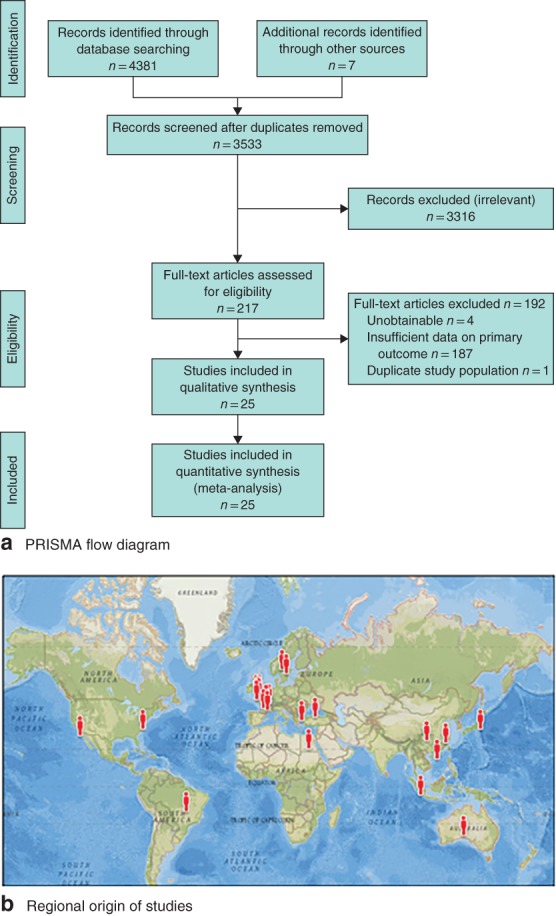
PRISMA flow diagram for the systematic review and regional origin of the studies. **a** PRISMA diagram; **b** map of regional origin of studies.

Of 4381 citations screened, some 25 studies comprising 4224 patients were included (*Fig*. 1 *a*). Of these patients, 2320 (54·9 per cent) and 1904 (45·1 per cent) underwent LTCE and LCD respectively. Three studies were RCTs; all others were retrospective studies. The publication date of the studies ranged from 1995 to 2016. The regional origin of the studies is displayed in *Fig*. 1 *b*. These studies are summarized in *Table* S2 (supporting information)^18^, ^19^, ^20^, ^21^, ^22^, ^23^, ^24^, ^25^, ^26^, ^27^, ^28^, ^29^, ^30^, ^31^, ^32^, ^33^, ^34^, ^35^, ^36^, ^37^, ^38^, ^39^, ^40^, ^41^, ^42^. The mean age of patients in the studies ranged from 38 to 68 years. The majority of studies (18 of 25) did not provide a breakdown of mean age per treatment arm. Only two studies^18^, ^19^ provided a breakdown of the male to female ratio for each treatment arm. None of the 25 studies reported mean BMI. Only one study^20^ reported the preoperative laboratory investigation (median (range) bilirubin concentration for LTCE 20 (6–74) μmol/l and for LCD 20 (7–89) μmol/l). Biliary colic was the most common patient presentation.

The median quality score for the RCTs was 8 (range 7–16) of 30, and that for the non‐randomized studies^14^ was 15 (range 10–27) of 40. Details of the quality scorings are provided in *Tables* S3 and S4 (supporting information).

**Figure 2:**
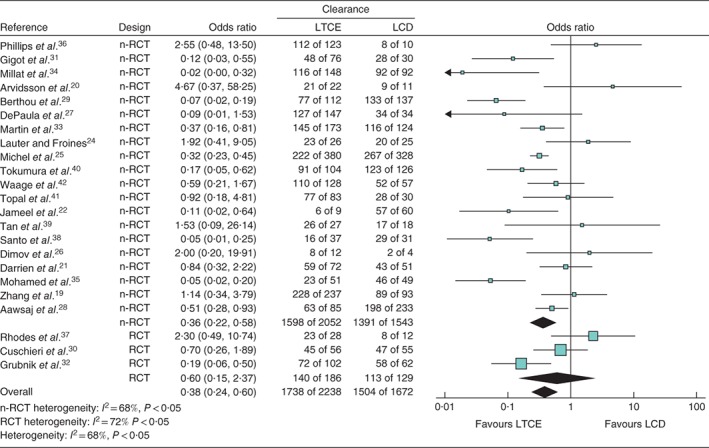
Forest plot for successful duct clearance in patients with choledocholithiasis undergoing a laparoscopic transcystic or transcholedochal approach. Studies that had 100 per cent success in both arms^18^, ^23^ were not included in the analysis, so calculation of an odds ratio was not possible in the pooled analysis. LTCE, laparoscopic transcystic exploration; LCD, laparoscopic choledochotomy; nRCT, non‐randomized clinical trial. A random‐effects model was used for meta‐analysis. Odds ratios are shown with 95 per cent confidence intervals.

**Figure 3:**
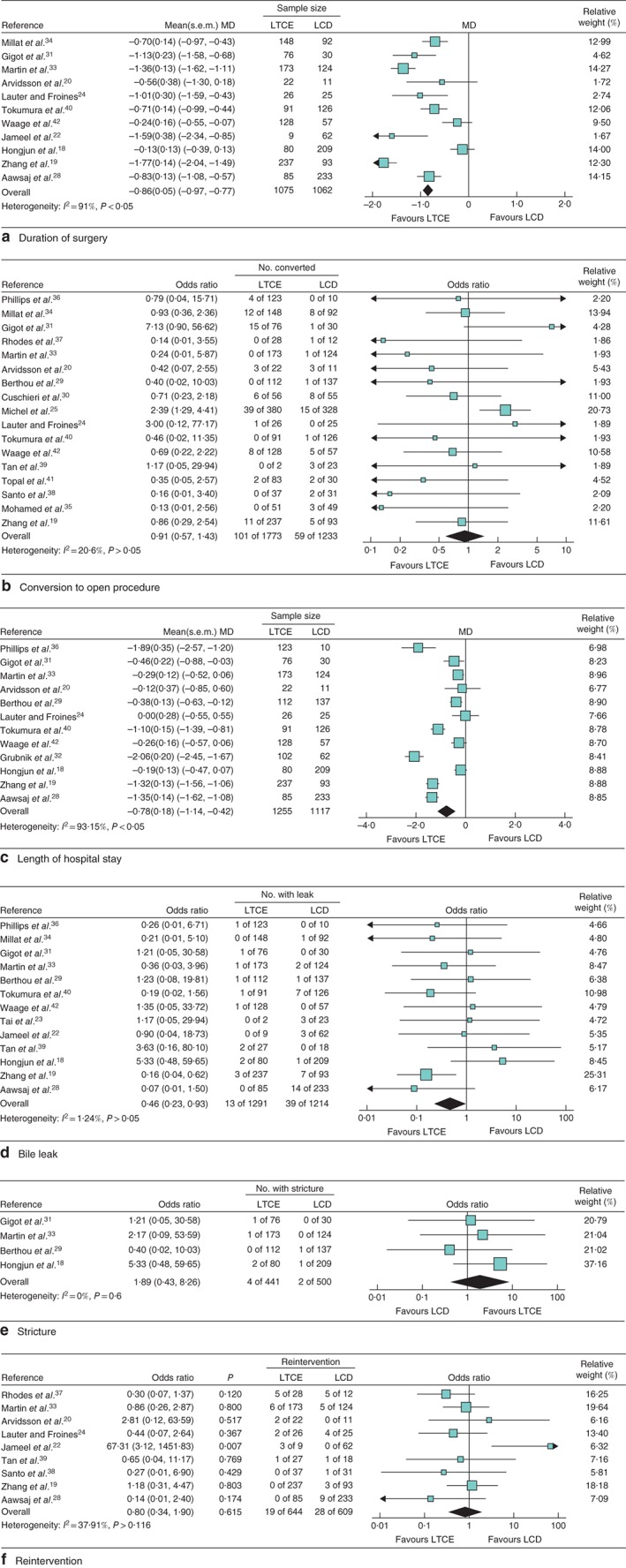
Forest plots for duration of surgery, conversion to open procedure, length of hospital stay, bile leak, stricture and reintervention in patients with choledocholithiasis undergoing a laparoscopic transcystic or transcholedochal approach. **a** Duration of surgery, showing an approximately 45 min longer operating time in the laparoscopic choledochotomy (LCD) group; **b** Conversion to open procedure; **c** length of hospital stay; **d** bile leak (as studies that had no leaks in either arm^20^, ^24^, ^25^, ^26^ were not included in generation of the forest plot, the software did not permit calculation of the odds ratio); **e** stricture; **f** reintervention (studies that reported no reinterventions^18^, ^26^, ^27^ were not included in the analysis, so calculation of an odds ratio was not possible). Random‐effects models were used for meta‐analysis. LTCE, laparoscopic transcystic exploration. **a,c** Standardized mean differences (MDs) and **b,d–f** odds ratios are shown with 95 per cent confidence intervals.

There was considerable heterogeneity regarding the primary outcome and the secondary outcomes of mean duration of surgery and hospital stay (*Figs* 2 and 3). The random‐effects model was therefore used for these outcome measures. There was non‐significant heterogeneity regarding conversion to an open procedure, stricture, bile leak and reintervention, yet, given the nature of the included studies, the random‐effects model was used^43^.

### Success rate

In all studies success was defined as complete duct clearance. The use of completion cholangiography to confirm duct clearance for both groups was clearly stated in eight of the 25 studies. One further study^30^ clearly stated that completion cholangiography was used in the LCD group but did not mention whether it was used in the LTCE group. Four other studies^19^, ^20^, ^33^, ^34^ stated that postoperative cholangiography was performed in patients undergoing biliary drainage to confirm clearance before removal of the T‐tube. Performance of completion cholangiography was not recorded in 11 studies. No significant association between instruments used and success was observed for either approach (*Table* S5, supporting information).

The odds of successful duct clearance were lower for LTCE than for LCD (OR 0·38, 95 per cent c.i. 0·24 to 0·59). No difference between the two approaches (OR 0·60, 0·15 to 2·37) was observed in RCTs, whereas the pooled estimate for non‐randomized trials showed significantly higher odds in favour of LCD (OR 0·36, 0·22 to 0·58).

### Cumulative analysis – temporal trend

The effect of time on outcome is shown in *Fig.* S1 (supporting information). From 1995 to 1999, no significant difference was observed between the two approaches. From 2000 onwards the studies consistently showed a higher rate of successful duct clearance with LCD compared with LTCE.

### Surgical data and morbidity

A shorter mean(s.d.) duration of surgery for LTCE compared with LCD (129(59) *versus* 175(61) min respectively; MD −0·86, 95 per cent c.i. −0·97 to −0·77) was observed (*Fig*. 3 *a*). No significant difference in conversion rate was found between the two approaches (*Fig*. 3 *b*). A significantly shorter mean hospital stay was seen for LTCE compared with LCD (MD −0·78, −1·14 to −0·42 (*Fig*. 3 *c*).

LTCE resulted in significantly fewer bile leaks than LCD (OR 0·46, 95 per cent c.i. 0·23 to 0·93) (*Fig*. 3 *d*). None of the RCTs reported on the incidence of bile leak in both arms. The incidence of biliary stricture did not significantly differ between the groups (*Fig*. 3 *e*).

No difference was seen in the pooled effect estimate for reintervention following for LTCE compared with LCD (OR 0·80, 95 per cent c.i. 0·34 to 1·90) (*Fig*. 3 *f*). *Table* S6 (supporting information) summarizes the types of procedure in each group after the primary intervention. From the aspect of patient selection, extracted data relating to the diameter of each duct, and the number and size of stones in each group were not informative (*Table* S7, supporting information).

### Publication bias

The classical fail‐safe N test of bias was significant (*Z* = −7·6, *P* < 0·001). The identified number of studies required for the *P* value to fall above α − α = 0·050 was 326 studies. A funnel plot demonstrating the distribution of standard error by the log odds ratio is shown in *Fig.* S2 (supporting information).

## Discussion

Successful duct clearance occurred more often with LCD than with LTCE. LCD was, however, associated with a longer duration of surgery and hospital stay. This was probably a result of the additional time required for sutured closure of the CBD and the higher risk of bile leak respectively. Bile duct suturing is a challenging task and has a significant learning curve^44^. Clipping the cystic duct stump is easier. No significant difference in conversion rate, bile duct stricture or reoperation was observed.

The included studies have recognized obstacles to successful LTCE, including an inability to negotiate the cystic duct (in particular due to long, tortuous cystic ducts with low insertions), multiple small stones in the non‐dilated CBD, and some stones being too large to be removed by LTCE^21^. It can be difficult and time‐consuming to remove multiple small stones using LTCE, with a significant risk of displacing some stones into the proximal CBD that cannot then be retrieved.

A temporal trend over time was observed. Studies published from 2000 all showed consistently higher odds of successful duct clearance with LCD. This was probably associated with improved technology, including the widespread use of high‐definition cameras and dedicated instruments. The refinement in surgical techniques and the learning curve could also have been a factor^44^, yet none of the included studies reported this being an issue. Before 2000 completion cholangiography was typically reserved for patients requiring biliary drainage, whereas after 2000 completion cholangiography appears to have been used more liberally to confirm stone clearance. It is possible that this may, in part, help explain the observed temporal trend in clearance rates.

The temporal trend observed does not negate the need for further studies to address this issue. Most studies included were not of high quality, with only three RCTs with significant heterogeneity. Scrutinizing the types and combination of instruments used did not reveal any clear pattern or difference between the two arms that may have accounted for the temporal trend seen before and after 2000.

In a single previous meta‐analysis^45^ on this subject, no significant differences between LTCE or LCD in the analysis of rates of stone clearance, conversion to open procedures, total morbidity, operating time or blood loss were observed. The authors observed a reduction in biliary complications in the LTCE group and concluded that this route was safer than the LCD approach. These results and conclusions are different to findings in the present study. This may be a result of the inclusion of studies with comparisons other than LCD *versus* LTCE and possible confusion in defining transcystic bile duct exploration.

Unfortunately, insufficient data were available in the included studies to make inferences about the impact on stricture formation of the relationship between duct diameter and the approach used. The authors are concerned, however, that there might be selection bias, with larger stones tending to be approached via the CBD. More recent trends towards primary closure without a T‐tube^22^ are often limited by the diameter of the bile duct as a risk factor for the leak^46^. The leak, however, is usually of little clinical importance compared with the added morbidity associated with T‐tube or stent insertion^47^.

There are limitations to this study. The full text of four relevant articles^48^, ^49^, ^50^, ^51^ could not be obtained, and there was not enough information in the abstracts on the primary outcome. By design, this study was liable for publication bias^52^. In an attempt to minimize this risk of bias, the inclusion of articles was not limited by language or date. In addition, the consistency of reporting of the secondary outcomes in the included papers was highly variable.

## Supporting information


**Table S1 Search strategies and Boolean characters used across the various databases**
**Table S2 Baseline demographics of included patients and summary of their presentations**
**Table S3 Quality assessment of included RCTs according to the Cochrane Handbook of Systematic Reviews** 42
**Table S4 Quality assessment of non‐RCTs included in the review according to the scoring system of West *et al*.** 43
**Table S5 Instrument details and completion cholangiography**
**Table S6 Summary of reinterventions in each group after the primary exploration and cholecystectomy**
**Table S7 Number of stones and duct diameter**
**Fig. S1 Cumulative analysis demonstrating the temporal trend for the primary outcome**
**Fig. S2 Funnel plot of standard error *versus* log odds ratio for statistical testing of publication bias**
