# Supplementary material for: Meta‐analysis of laparoscopic transcystic versus transcholedochal common bile duct exploration for choledocholithiasis
Source: BJS Open. 2019 Jan 23;3(3):242–51. doi: 10.1002/bjs5.50132 (PMC6551404; doi:10.1002/bjs5.50132)

**BJS5_50132**

**Meta-analysis of laparoscopic transcystic *versus* transcholedochal common bile duct exploration for choledocholithiasis**

**M. Bekheit, R. Smith, G. Ramsay, F. Soggiu, M. Ghazanfar and I. Ahmed**

**Table S1 Search strategies and Boolean characters used across the various databases**

| Database | Search Type | Limits | Search Terms Used |
| --- | --- | --- | --- |
| Cochrane | Advanced | Nil | (Transcystic OR Choledochal OR CBD)  “AND”  (Laparoscopic OR open OR convention OR celioscopic)  “AND”  (stone OR lithiasis OR gall stone OR CBD stone) |
| Centre for controlled trial Registers | Advanced | Nil | (Transcystic OR Choledochal OR CBD)  “AND”  (Laparoscopic OR open OR convention OR celioscopic)  “AND”  (stone OR lithiasis OR gall stone OR CBD stone) |
| Web of Science | Advanced | Nil | (Transcystic OR Choledochal OR CBD)  “AND”  (Laparoscopic OR open OR convention OR celioscopic)  “AND”  (stone OR lithiasis OR gall stone OR CBD stone) |
| Trip | Advanced | Nil | (Transcystic OR Choledochal OR CBD)  “AND”  (Laparoscopic OR open OR convention OR celioscopic)  “AND”  (stone OR lithiasis OR gall stone OR CBD stone) |
| PubMed | Advanced | Nil | (Transcystic OR Choledochal OR CBD)  “AND”  (Laparoscopic OR open OR convention OR celioscopic)  “AND”  (stone OR lithiasis OR gall stone OR CBD stone) |
| Ovid | Advanced | Nil | (Transcystic OR Choledochal OR CBD)  “AND”  (Laparoscopic OR open OR convention OR celioscopic)  “AND”  (stone OR lithiasis OR gall stone OR CBD stone) |
| Embase | Advanced | Nil | (Transcystic OR Choledochal OR CBD)  “AND”  (Laparoscopic OR open OR convention OR celioscopic)  “AND”  (stone OR lithiasis OR gall stone OR CBD stone) |

**Table S2 Baseline demographics of included patients and summary of their presentations**

| Study | Total Number of Patients | Age LTCE* vs LCD** (Years) Mean (range) | Sex (Male %) | Cholecystitis **%** | Cholangitis % | Jaundice % | Pancreatitis % | Colic % | Success of LTCE vs LCD  Odds ratio & *p value* |
| --- | --- | --- | --- | --- | --- | --- | --- | --- | --- |
| Hongjun ^18^ | 289 | 60*** | 65.4 | - | - | - | - | - |  |
| Zhang ^19^ | 330 | 54.7*** vs 54.2*** | 42.5 | 11 | - | - | - | - | 1.14/<0.05 |
| Arvidsson ^20^ | 33 | 38 (21-81) vs 38 (20-67) |  | 10.3 | - |  | 5.1 | - | 4.67/<0.05 |
| Darrien ^21^ | 123 | (14 – 92)^ |  | 10 | 9.7 | 7.9 | 10.7 | 55 | 0.84/<0.05 |
| Jameel ^22^ | 69 | 62 (29-87) vs 71 (19-90) |  | 29.6 | 8.5 | 22.2 | 14 | 15.5 | 0.11/<0.05 |
| Tai ^23^ | 25 | 60 (16-84) | 95.9 | - | - | - | - | - |  |
| Lauter ^24^ | 51 |  |  | - | - | - | - | - | 1.92/<0.05 |
| Michel ^25^ | 708 | 61 (17 – 91) |  | 17.3 | 15.4 | 12.6 | 3.3 | 63 | 0.32/<0.05 |
| Dimov ^26^ | 16 | 60.5*** | 16.7 | - | - | - | - | - | 2/>0.05 |
| DePaula ^27^ | 181 | 52 (17 – 90) | 37.6 | - | - | - | - | - | 0.09/>0.05 |
| Aawsaj ^28^ | 318 | 60 (16 – 84) vs 58 (16 – 18) | 31.4% | - | - | - | - | - | 0.51/<0.05 |
| Berthou ^29^ | 249 | 63 (19-93) | 26.8% | 43.2 | 14.1 | 29.1 | 6.4 | 71.8 | 0.07/<0.05 |
| Cuschieri ^30^ | 111 | (18 – 89)^ vs (19-88)^ | 34% | - | - | 58.7 | 6.7 | 93 | 0.69/<0.05 |
| Gigot ^31^ | 106 | 62 (17 - 86) | 32.6 | - | - | - | - | - | 0.12/<0.05 |
| Grubnik ^32^ | 164 | 61.9 (27 – 81) | 16.4 | 33.6 | - | 22.7 | 22.3 | - | 0.17/<0.05 |
| Martin ^33^ | 297 | 48*** vs 56*** |  | - | - | - | - | - | 0.36/<0.05 |
| Millat ^34^ | 240 | 68 (21-92) |  | 36 | 20.3 | 33.6 | 10.5 | 44.1 | 0.02/>0.05 |
| Mohamed ^35^ | 100 | 47.1 (34-72) | 17.3 | - | - | 4.7 | 7.3 | - | 0.05/<0.05 |
| Phillips ^36^ | 133 | 41 (20 – 81) |  | - | - | - | - | - | 2.55/<0.05 |
| Rhodes ^37^ | 40 | 62 (28 – 83) | 2.5 | - | - | - | - | - | 2.3/<0.05 |
| Santo ^38^ | 68 | 51.6 (20 – 85) | 24.3 | - | - | - | - | - | 0.05/<0.05 |
| Tan ^39^ | 45 | 60 (27 – 85) | 32 | 10 | 32 | 46 | 10 | 60 | 1.53/>0.05 |
| Tokumura ^40^ | 230 | 66.4*** | 46.5 | - | - | - | - | - | 0.17/<0.05 |
| Topal ^41^ | 113 | 62 *** vs 76*** | 24.8 | 21.2 | - | - | - | - | 0.92/<0.05 |
| Waage ^42^ | 185 | 49 (20 – 83) |  | - | - | - | - | - | 0.59/<0.05 |
| *CD: Cystic duct, **BD: bile duct, *** no range reported, ^ no mean reported. | | | | | | | | | |

**Table S3 Quality assessment of included RCTs according to the Cochrane Handbook of Systematic Reviews^42^**

|  | Sequence Generation | Allocation concealment | Blinding | Incomplete data outcomes | Selective outcome reporting | Baseline imbalance | Total |
| --- | --- | --- | --- | --- | --- | --- | --- |
| Cuschieri ^30^ | 3 | 3 | 1 | 3 | 3 | 3 | 16 |
| Grubnik ^32^ | 1 | 1 | 2 | 1 | 1 | 2 | 8 |
| Rhodes ^37^ | 1 | 1 | 2 | 1 | 1 | 1 | 7 |

**Table S4 Quality assessment of non-RCTs included in the review according to the scoring system of West *et al*.^43^**

| Study | Question definition | Intervention definition | Outcome definition | Sampling population | Confounders considered in Stat | Multifactor testing | measure of precision | bias and limitations | Conclusion supported by results | Total |
| --- | --- | --- | --- | --- | --- | --- | --- | --- | --- | --- |
| Zhang ^19^ | 3 | 2 | 3 | 5 | 1 | 3 | 3 | 1 | 3 | 24 |
| Santo ^38^ | 2 | 2 | 1 | 2 | 1 | 1 | 1 | 1 | 2 | 13 |
| Arvidsson ^20^ | 2 | 2 | 2 | 3 | 1 | 1 | 1 | 1 | 2 | 15 |
| Aawsaj ^28^ | 2 | 2 | 2 | 3 | 1 | 1 | 1 | 1 | 2 | 15 |
| Berthou ^29^ | 2 | 2 | 3 | 2 | 1 | 1 | 1 | 1 | 1 | 14 |
| Darrien ^21^ | 3 | 3 | 3 | 3 | 1 | 1 | 1 | 1 | 2 | 18 |
| DePaula ^27^ | 3 | 3 | 2 | 2 | 1 | 1 | 1 | 1 | 1 | 15 |
| Dimov ^26^ | 1 | 1 | 1 | 2 | 1 | 1 | 1 | 1 | 1 | 10 |
| Gigot ^31^ | 3 | 3 | 3 | 5 | 3 | 3 | 3 | 2 | 2 | 27 |
| Jameel ^22^ | 2 | 1 | 2 | 2 | 1 | 1 | 1 | 1 | 2 | 13 |
| Lauter ^24^ | 2 | 2 | 2 | 3 | 1 | 1 | 1 | 1 | 2 | 15 |
| Martin ^33^ | 2 | 2 | 2 | 3 | 1 | 1 | 1 | 1 | 2 | 15 |
| Michel ^25^ | 2 | 2 | 3 | 3 | 2 | 2 | 2 | 2 | 2 | 20 |
| Millat ^34^ | 2 | 1 | 1 | 2 | 1 | 1 | 1 | 1 | 2 | 12 |
|  |  |  |  |  |  |  |  |  |  |  |
| Mohamed ^35^ | 2 | 2 | 2 | 2 | 1 | 1 | 1 | 1 | 2 | 14 |
| Phillips ^36^ | 2 | 2 | 3 | 3 | 1 | 1 | 1 | 1 | 2 | 16 |
| Tai ^23^ | 2 | 1 | 1 | 3 | 1 | 1 | 1 | 1 | 1 | 12 |
| Tan ^39^ | 2 | 1 | 1 | 5 | 1 | 1 | 1 | 1 | 1 | 14 |
| Tokomura ^40^ | 2 | 1 | 2 | 3 | 1 | 1 | 1 | 2 | 2 | 15 |
| Topal ^41^ | 3 | 3 | 3 | 5 | 3 | 3 | 3 | 2 | 2 | 27 |
| Waage ^42^ | 2 | 3 | 3 | 5 | 1 | 1 | 2 | 1 | 2 | 20 |
| Hongjun ^18^ | 3 | 3 | 3 | 3 | 3 | 2 | 2 | 2 | 2 | 23 |

**Table S5 Instrument details and completion cholangiography**

| Study | Instrument Details CD | Instrument Details CBD | **Cholangiography** | **Comment** |
| --- | --- | --- | --- | --- |
| Hongjun ^18^ | Water flush  Wire Basket | Wire basket | No |  |
| Zhang ^19^ | Dormia basket | Electrohydraulic lithotripsy  Dormia basket  T-tube | Yes – in cases of biliary drainage | Patients in the T-tube group had a cholangiogram on the 3^rd^- to 5th postoperative day |
| Santo ^38^ | Saline flush  Balloon dilation  Basket extraction | Saline flush  Balloon dilation  Basket extraction | No |  |
| Arvidsson ^20^ | Balloon catheter  Wire basket | Balloon catheter  Wire basket  T-tube | Yes – in cases of biliary drainage | Cholangiogram done on 7th-12th post op day in cases were T-tube used – before removal |
| Aawsaj ^28^ | 3mm Choledochoscope | 5mm Choledochoscope  T-tube | No |  |
| Berthou ^29^ | Wire basket  Fogarty balloon | Wire basket  Fogarty balloon | No |  |
| Darrien ^21^ |  |  |  |  |
| DePaula ^27^ | Wire basket  Balloon  Grasper | Wire basket  Balloon  Grasper | No |  |
| Dimov ^26^ | - | - | Yes | Clear statement that CC used to confirm stone clearance |
| Gigot ^31^ | 6-F Dormia basket  Angioplasty balloon catheter | Blind Dormia basket Forgati balloon catheter | Yes | Clear statement that duct clearance confirmed by CC |
| Grubnik ^32^ | 3mm Flexible choledochoscope.  Dorma Basket (primarily)  Balloon Dilatation  Trancystic biliary Drain in (some cases) | Choledochoscope  Stones retrieved with tramatic forceps,  Blunt forceps,  Saline irrigation Dorma Basket  Electrohydraulic lithotripsy (difficult cases)  T-tube in some cases | Yes | Clear statement that CC performed |
| Jameel ^22^ | Wire basket | Berci knife and microscissors  Wire basket  T-tube | Yes | Clear statement that duct clearance confirmed by CC |
| Lauter ^24^ | Saline flush, glucagon  Basket extraction | - | Yes | Clearly documented in table |
| Martin ^33^ | Saline flushing + glucagon  4.5mm ureteric stone basket | Balloon catheter  Dorma Basket  T-tube | Yes – in cases of biliary drainage | Clear statement that CC used during follow-up in cases of biliary drainage |
| Michel ^25^ | Dorma Basket  Balloon Catheter | Choledochoscope | No |  |
| Millat ^34^ | Choledochoscope  Dorma Basket extraction  Wire basket | Choledochoscope | Yes – in cases of biliary drainage | Statement that CC done in cases of Biliary drainage |
| Mohamed ^35^ | Choledochoscope | Choledochoscope  Dorma Basket  T tube | No |  |
| Tai ^23^ | Saline flushing  Glucagon injection | Stone forceps,  saline flushing,  dormia basket,  balloon catheter | Yes | States that duct clearance based on radiological findings - including completion cholangiogram |
| Tan ^39^ | 2.8 mm Choledochoscope  Nathanson Basket  Zero tip Nitinol Basket | 5 mm Choledochoscope  Basket extraction  T-tube/ interbiliary stent | Yes | Clearly states that completion cholangiogram performed |
| Tokumura ^40^ | Balloon  Flexible 2.8-mm Choledochoscope | Choledochoscope (5 or 6mm)  Basket | Yes | Clear statement that duct clearance confirmed by CC |
| Topal ^41^ | Nitinol stone retrieval basket | Nitinol stone retrieval basket | No |  |
| Cuschieri ^30^ |  |  |  |  |
| Phillips ^36^ |  |  |  |  |
| Rhodes ^37^ |  |  |  |  |
| Waage ^42^ | 3mm - Choledochoscope  Balloon catheter  Wire stone basket | 5 mm Choledochoscope  T tube | Yes | Clear statement that completion cholangiogram done for BD group |

**Table S6 Summary of reinterventions in each group after the primary exploration and cholecystectomy**

| Study | Re-intervention Details |  |
| --- | --- | --- |
|  | CD | BD |
| Zhang ^19^ | 9 Further Procedures  9 x Endoscopic Sphincterotomy/ Papillary balloon dilatation | 3 Further Procedures  3 x Endoscopic Sphincterotomy |
| Santo ^38^ | 0 Further Procedures | 1 Further Procedure  Re-laparoscopy for toxaemia and output of biliary secretions through penrose drain – fistula of suture of choledocus |
| Arvidsson ^20^ | 2 Further Procedures  1 x submucous dissection of the CD and part of CBD due to faulty technique in dilating the cystic duct. A transcystic drain was left and gallbladder removed. Patient underwent laparotomy three days later Because of increasing jaundice. The partially damaged lateral wall of the duct was closed around a T – tube, which was removed after 9 weeks  1 x laparoscopy for arterial bleeding from the liver bed | 0 Further Procedures |
| Aawsaj ^28^ | 0 Further Procedures | 9 Further Procedures  2x patients returned to theatre for post-op bleeding  4x Laparoscopy + subsequent ERCP for bile leak (due to diagnostic uncertainty)  3x ERCP directly with a biliary stent placed for bile leak |
| Jameel ^22^ | 3 Further Procedures  3 x Postoperative ERCP | 0 Further Procedures |
| Lauter ^24^ | 2 Further Procedure  2 x ERCP for retained Stones | 4 Further Procedure  3 x ERCP for retained stones  1x Failed ERCP with Interventional Radiology for retained stone |
| Martin ^33^ | 6 Further Procedures:  1 x Laparoscopy to reclip cystic artery Haemorrhage  1x Laparoscopic T-tube replacement for dislodged T-tube  1x Laparotomy and drain insertion for Peritonitis on T tube removal  1x Laparoscopy for T-tube removal and drainage for T-tube that could not be removed  1x Open Roux-en-Y hepaticojejunostomy for pain and poor biliary drainage  1 x Open Roux-en-Y choledochojejunostomy for Biliary Stricture | 5 Further Procedures:  1 x Laparoscopic Resuturing for bile leak  1x Laparoscopic removal of retained stone, antegrade stent and resuturing for bile leak  1x Laparoscopic ERCP and resuturing for worsening jaundice and stich occluding duct  1 x ERCP with stent and laparoscopic washout for bile ascities  1x Failed ERCP, laparoscopic removal of stent and insertion of T-tube for worsening jaundice and stent not in duodenum |
| Rhodes ^37^ | 5 Further Procedures:  1 x Laparoscopic stent (Replacement of biliary endoprosthesis for bile leak) + postoperative ERCP  4 x Postoperative ERCP | 5 Further Procedures:  1 x Laparotomy for Bile Leak (open ECBD)  4 x Laparoscopic stent plus postoperative ERCP |
| Tan ^39^ | 1 Further Procedure  1x ERCP + insertion of stent + percutaneous drainage for bile leak and sub-hepatic abscess | 1 Further Procedure  1x Percutaneous drainage for intra-abdominal abscess |

**Table S7 Number of stones and duct diameter**

|  | CD | | | | BD | | | |
| --- | --- | --- | --- | --- | --- | --- | --- | --- |
| Study | Stone Number  Mean (Range) | Stone Size  Mean (Range) mm | Cystic Duct Diameter  Mean (Range) mm | Common Bile duct diameter Mean (Range) mm or (+/- SD) | Stone Number  Mean (Range) | Stone Size  Mean (Range) mm | Cystic Duct Diameter  Mean (Range) mm | Common Bile duct diameter Mean (Range) mm or (+/- SD) |
| Hongjun ^18^ | - | - | 0.47 (+/- 0.09) | 1.04 (+/- 0.24) | - | - | 0.47 (+/- 0.08) | 1.18 (+/- 0.29) |
| Zhang ^19^ | 3.2 ± 1.8 | 5.3 ± 2.1 | - | - | 4.5 (± 2.6) | 12.0 (± 3.5) | - | - |
| Grubnik ^32^ | 1 (1-7) | - | - | 8.5 (6-10) | 2.5 (1-40) | - | - | 10.5 (6-20) |
| Michel ^25^ | 1.9 (1-6) | - | - | 7.8 (5-10) | 4.5 (1-28) | - | - | 12.4 (7-20) |

**Fig. S1 Cumulative analysis demonstrating the temporal trend for the primary outcome**


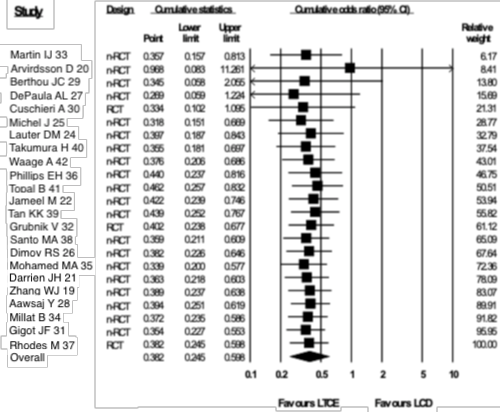


**Fig. S2 Funnel plot of standard error *versus* log odds ratio for statistical testing of publication bias**


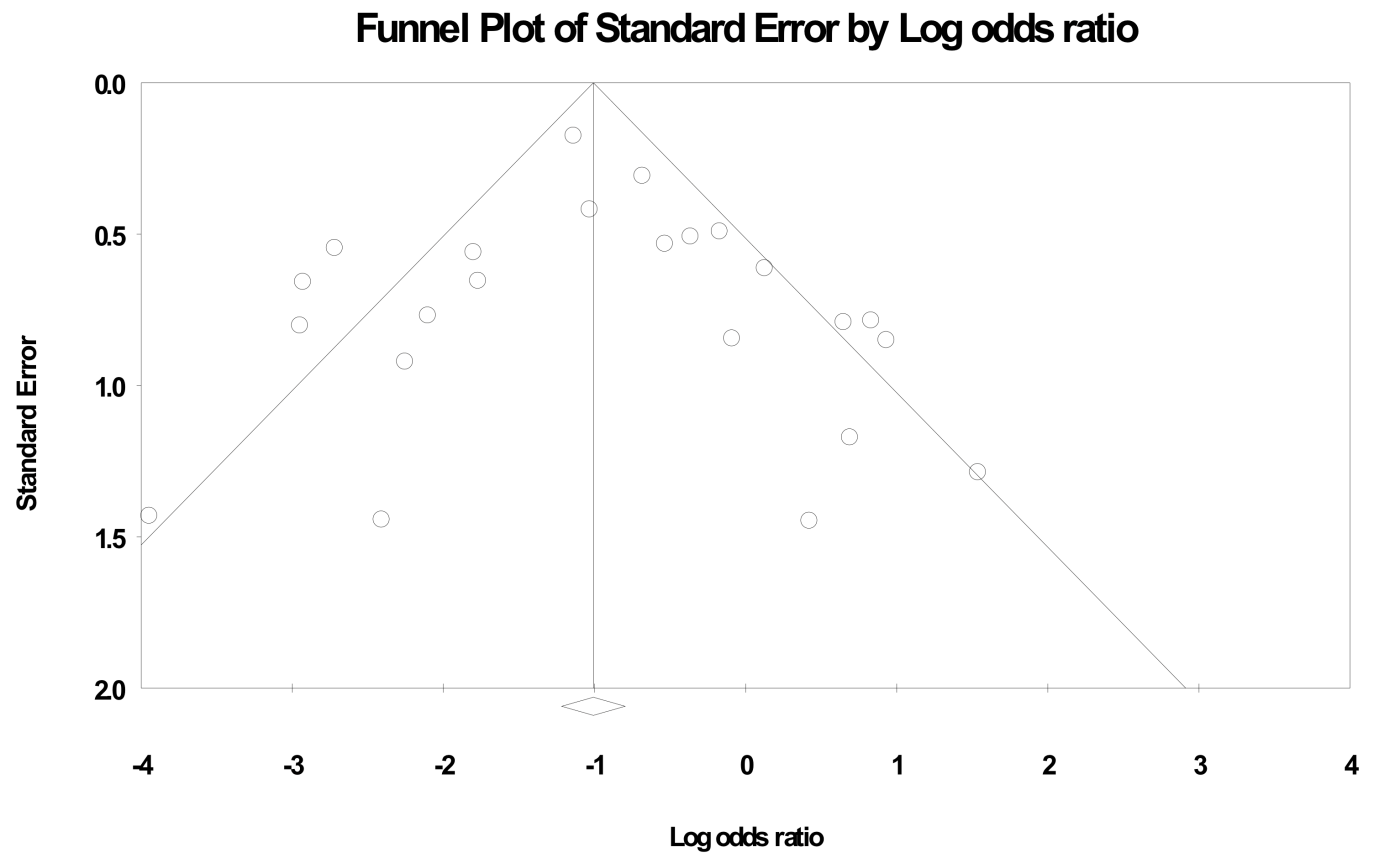

Supplement: Supplementary file 1 — Table S1 Search strategies and Boolean characters used across the various databases Table S2 Baseline demographics of included patients and summary of their presentations Table S3 Quality assessment of included RCTs according to the Cochrane Handbook of Systematic Reviews 42 Table S4 Quality assessment of non‐RCTs included in the review according to the scoring system of West et al. 43 Table S5 Instrument details and completion cholangiography Table S6 Summary of reinterventions in each group after the primary exploration and cholecystectomy Table S7 Number of stones and duct diameter Fig. S1 Cumulative analysis demonstrating the temporal trend for the primary outcome Fig. S2 Funnel plot of standard error versus log odds ratio for statistical testing of publication bias [file BJS5-3-242-s001.docx]
